# Stress, genomic adaptation, and the evolutionary trade-off

**DOI:** 10.3389/fgene.2014.00092

**Published:** 2014-04-23

**Authors:** Steven D. Horne, Saroj K. Chowdhury, Henry H. Q. Heng

**Affiliations:** ^1^Center for Molecular Medicine and Genetics, School of Medicine, Wayne State UniversityDetroit, MI, USA; ^2^John D. Dingell VA Medical CenterDetroit, MI, USA; ^3^Department of Pathology, School of Medicine, Wayne State UniversityDetroit, MI, USA

**Keywords:** genome theory, genome instability, chromosomal instability, stress response, somatic evolution

## Abstract

Cells are constantly exposed to various internal and external stresses. The importance of cellular stress and its implication to disease conditions have become popular research topics. Many ongoing investigations focus on the sources of stress, their specific molecular mechanisms and interactions, especially regarding their contributions to many common and complex diseases through defined molecular pathways. Numerous molecular mechanisms have been linked to endoplasmic reticulum stress along with many unexpected findings, drastically increasing the complexity of our molecular understanding and challenging how to apply individual mechanism-based knowledge in the clinic. A newly emergent genome theory searches for the synthesis of a general evolutionary mechanism that unifies different types of stress and functional relationships from a genome-defined system point of view. Herein, we discuss the evolutionary relationship between stress and somatic cell adaptation under physiological, pathological, and somatic cell survival conditions, the multiple meanings to achieve adaptation and its potential trade-off. In particular, we purposely defocus from specific stresses and mechanisms by redirecting attention toward studying underlying general mechanisms.

## UNIFYING THE WIDE VARIETY OF CELLULAR STRESSES

Under either normal physiological or pathological conditions, cells are subject to a wide variety of internal and external stresses, which have been associated with a variety of biological responses. For example, responses to endoplasmic reticulum (ER) stress include cell death, inflammatory signaling, insulin resistance, and lipogenesis (Kaufman, 1999; Schroder, 2008; Zhang and Kaufman, 2008; Lee et al., 2012). Exposure to ROS stress can result in transient growth arrest, increase in cellular proliferation, permanent growth arrest or senescence, and cell death (Pelicano et al., 2004). While ongoing efforts are being placed on identifying stress-associated molecular mechanisms and their interactions (Rutkowski and Hegde, 2010; Walter and Ron, 2011; Hetz, 2012) and linking their contributions to system homeostasis and many common diseases, the complexity, heterogeneity, and combinations of these stresses and cellular responses can challenge the characterization of a specific gene’s or pathway’s role in disease onset and progression.

Despite gaining deeper understanding regarding each specific stress response pathway, the introduction of various large-scale omics technologies has provided conflicting information in understanding functions of individual pathways in the entire system context (Heng et al., 2009, 2011a; Abu-Asab et al., 2011). The cellular stress response is a reaction to any form of macromolecular damage that exceeds a set threshold, independent of the underlying cause, and the fragmented knowledge of the stress response needs to be unified at the conceptual level to explain its universality for many different species and types of stress (Kultz, 2003). In fact, many aspects of the cellular stress response are not stressor-specific, because cells monitor stress based on macromolecular damage without regard to the type of stress that causes such damage (Kultz, 2005). There is also limited pathway specificity for stress response during somatic cell evolution, especially under pathological conditions where stochastic genetic alteration plays an important role.

To establish a common mechanism of stress response, rather than continuing to link more genes to different pathways by studying gene regulations and interactions in more linear experimental models, research efforts need to be focused on the genome dynamics during somatic cell evolution, as the stress response represents a key component of somatic cell evolution, impacting on many physiological and disease conditions (Heng et al., 2011b, 2013a). To achieve this goal, two major changes are needed. First, we need new strategies to monitor the stress response at the cellular system level. Despite source and degree variance, stress clearly results in system change. Thus, we will generalize stress to encompass the wide variety of internal and external stressors and pathways, as increased system dynamics is the common consequence. This holistic approach can provide understanding regarding the impact of stress to the cellular system and its implications to common disease without attempting to decipher massive amounts of potentially conflicting molecular data. Second, in contrast to the misconception that stress is bad and the stress-response mainly is a means to overcome “negative” influence, the stress response is essential for biological function. ER stress is required in B cell lymphopoiesis (Zhang et al., 2005), certain degrees of hypoxic stress can increase the homing of tissue-specific stem cells, and stress-induced genome variations are important for short-term evolutionary adaptation (Heng et al., 2013b). While the stress response is essential for life by creating heterogeneity-mediated robustness, it also generates biological damage for the system, in particular, when stress is high. These damages represent the trade-off to adapt under stress.

Clearly, to study the general stress response mechanism, the appropriate evolutionary framework is needed. Since many reviews have discussed gene- and pathway-specific mechanisms, we will focus on the genome perspective.

## STRESS INDUCES SYSTEM DYNAMICS AT MULTIPLE LEVELS

Currently, most molecular characterization of stress focuses on the gene and pathway levels. Great progress in the field has achieved the understanding of the regulatory mechanisms and signaling crosstalk of the three branches of the unfolded protein response (UPR; Hetz, 2012). It is known that ER stress is buffered by the activation of UPR, and failure to adapt to ER stress leads to apoptosis. Increased studies revealed many layers of interaction/crosstalk and molecular heterogeneity. Many novel physiological outcomes of the UPR that are not directly related to protein-folding stress have been discovered, including metabolism, innate immunity, cell differentiation, functional composition, and somatic cell evolution. Many diseases with different molecular mechanisms are also linked to ER stress, further complicating this issue.

Since genetic organization can be divided into gene and genome levels, and genome-level alteration plays a key role in cancer evolution (Heng, 2009; Heng et al., 2011a,b), it is necessary to investigate the often-ignored linkage between ER stress and genome aberrations. One interesting window is to study cell death-mediated karyotype changes. Many current researchers analyze how ER stress results in cell death, as if when apoptosis occurs, the story ends. When the results of cell death are under investigation, however, a new picture emerges: not all cells under ER stress-mediated cell death will die, some of them do survive, but display altered genomes (Stevens et al., 2007, 2010, 2011, 2013, 2014; Stevens and Heng, 2013; Liu et al., 2014). Furthermore, stress in general, even before reaching the point of cell death, results in many infrequent genome alterations (Heng et al., 2006).

These seemingly random non-clonal chromosome aberrations (NCCAs), encompassing all random structural and numerical aberrations, have been ignored as insignificant genetic noise. However, elevated NCCA frequency represents increased genome-level system dynamics and can be linked to virus infection, drug treatment, many types of environmental stress, and tumorigenicity (Ye et al., 2009; Heng et al., 2013a).

**FIGURE 1:**
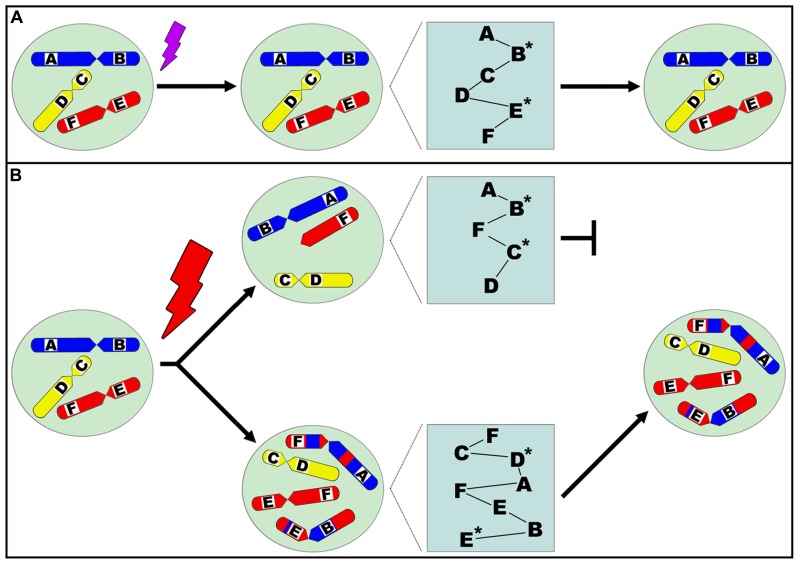
**Diagram illustrating the relationship between stress, genome topology alteration, resulting genetic network reorganization, and successful evolutionary selection.** Different chromosomes are designated by color (red, yellow, blue) and drawn within the nucleus, representing the genome, and genes are designated A, B, C, D, E, F within the chromosomes. Corresponding protein networks are illustrated by the relationships between proteins A, B, C, D, E, F. A cell is exposed to a moderate level of stress **(A)**, resulting in genetic and/or epigenetic alteration as indicated by asterisks (*) next to impacted proteins. The cell survives the stress event without genome-level alteration. When a cell is exposed to a high-level of stress **(B)**, this results in genome topology alteration represented by numerical aberrations (e.g., aneuploidy) and/or structural aberrations (e.g., translocations). This directly affects the physical three-dimensional relationship between genes and changes the overall genetic network structure, resulting in drastic systemic changes beyond the influence of genetic and/or epigenetic alterations that may concurrently occur. As a consequence, the corresponding protein network changes are shown by altered relationships between proteins. These new genomic systems then undergo evolutionary selection, and those that are stochastically selected upon may clonally expand and dominate the cell population.

Thus, the general mechanism of stress is to trigger alteration of system dynamics at multiple levels. In addition to the fact that whether or not a specific change is good or bad is context-dependent, the trade-off can be reflected at multiple levels in addition to cell death. Stress-response can be classified into three types. The first type is caused by a low-level of stress, resulting in increased non-genetic dynamics that only require an energy cost for recovery. The second type is caused by an intermediate level of stress, resulting in gene and/or epigenetic alteration. The third type is caused by the highest level of stress that can result in genome-level reorganization. In addition to the level of stress, the duration of the stressful condition also contributes to how multiple genetic and non-genetic factors respond. For example, long-term low-level stress could also trigger genome-level alterations. In general, under lower-levels of stress, system recovery can be achieved even though epigenetic and gene mutations may be involved. In contrast, high-levels of stress can lead to genome alteration, the point of no return for the individual cell. Lower-levels of stress often create stepwise evolutionary adaptation whereas high-level stress can lead to massive death or occasionally successful punctuated macroevolution. Finally, when the cell population is dominated by altered genomes, the disease will become obvious. Previous studies have supported that high-levels of stress can induce genome chaos, characterized by rapid, stochastic genome shattering and reorganization (Heng, 2007b, 2014; Liu et al., 2014). This results in network restructuring and rewiring, as evidenced by observed transcriptome elevation associated with karyotypic alteration (Stevens et al., 2014). Therefore, cells that survive this process display altered karyotypes and systems (**Figure 1**). Linking these different degrees of stress in this scheme would suggest that for a system to sense a particular stress, specificity might be less important than its degree or intensity. In addition, due to stochasticity, there may not be a specific response or end product to a particular stress or degree. This is especially important for disease research that focuses of the long-term consequences of stress, as stochastic genome variation has been associated with common disease.

## STOCHASTIC GENOME VARIATION IS ASSOCIATED WITH COMMON DISEASE AND WITHIN NORMAL TISSUE

The search for molecular causative mechanisms of common diseases has resulted in the identification of high-level genome alterations. Autism and Alzheimer’s disease are associated with altered karyotypes (Ye et al., 2007; Iourov et al., 2008). CGH analysis revealed that 80% of children with intellectual disability, epilepsy, autism, and congenital anomalies exhibited CNVs, chromosomal imbalances, or meiotic genome instability (Iourov et al., 2012a). Aneuploidy has been detected in several brain diseases (Iourov et al., 2012b). Stochastic genome alterations have been observed in Gulf War Illness and chronic fatigue syndrome patients (Heng et al., 2013b), and these diseases have been linked to elevated genome instability (Heng et al., unpublished data). Celiac and Crohn’s disease patients display significantly increased numbers of chromosomal aberrations in peripheral blood lymphocytes (Hojsak et al., 2013). Increased polyploidy was observed in cardiomyocytes associated with hypertension, cardiac overloading, and congenital heart disease (Davoli and de Lange, 2011).

This association suggests similarities to cancer (McClellan and King, 2010), where most cancer arises from stochastic genome alterations rather than common gene mutations (Heng et al., 2006; Heng, 2007c, 2010). Unlike single gene-driven disease, in which highly penetrant genetic defects are detectable within a patient population, the molecular evolution of most cancers can only be explained by the evolutionary mechanism that is equal to all molecular mechanisms in the entire patient population (Ye et al., 2009; Heng et al., 2010). It is also known that the *de novo* locus-specific rate of genomic rearrangement is at least 100- to 10,000-fold greater than the rate of point mutations (Lupski et al., 2010).

Surprisingly, genome alterations have been reported in normal, healthy tissues, including the polyploidization of liver cells, skeletal muscle, and Purkinje neurons, as well as blastocyst mosaicism and trisomy 21 mosaicism in the general population (Celton-Morizur and Desdouets, 2010; Davoli and de Lange, 2011; Fragouli and Wells, 2011; Hulten et al., 2013). An increase of genome-level alterations in healthy individuals has been revealed by whole-genome sequencing application (Abecasis et al., 2012). Chromosomal aneuploidy, chromosome non-disjunction, and micronuclei formation in peripheral lymphocytes are associated with age (Ohshima and Seyama, 2010). Polyploidy increases with age in hepatocytes (Gentric et al., 2012). Somatic mosaicism as a result of chromosome instability and aneuploidy has been proposed to play a role in brain aging (Faggioli et al., 2011; Iourov et al., 2012b).

What is the difference between normal and disease tissue in terms of genome alterations? Overall, in pathological conditions, the frequencies of stochastic genome change are elevated and coupled with the presence of specific clonal chromosome aberrations (CCAs). In addition, the degree of genome alteration is much higher for each cell.

## GENOME THEORY OFFERS EXPLANATIONS

To explain the widely detected stochastic genome alterations in normal and disease conditions, a new framework is needed, as current gene theory fails to achieve satisfactory explanations. Gene theory states that DNA sequence serves as the genetic blueprint, where information transfers from DNA to RNA to proteins. Accordingly, defective genes are the main cause of disease and should be readily identifiable. However, defective genes are rarely the common drivers of disease when considering the large number of essential genes, and only under very specific circumstances does this concept hold true, as in the cases of sickle cell anemia and chronic phase chronic myeloid leukemia (Horne et al., 2013b). Furthermore, personal whole-genome sequencing revealed high numbers of gene mutations for healthy individuals, illustrating disconnect between gene mutation and most common diseases (Abecasis et al., 2012).

In contrast, the recently introduced genome theory calls for a shift from the gene to the genome, as genes and genomes represent different levels of genetic organization with distinct coding systems (Heng, 2009; Heng et al., 2009, 2011a). The information regarding assembly of parts is most likely not stored within the individual gene or genetic locus. DNA only encodes for the parts and some tools of the system (RNAs, proteins, regulatory elements). The complete interactive genetic network is coded by genome topology-mediated self-organization (Ye et al., 2007; Heng, 2009, 2010; Heng et al., 2010, 2011a). The genome is not merely the entire DNA sequence or the vehicle of all genes. Rather, the genome context or landscape (genomic topologic relationship among genes and other sequences within three-dimensional nuclei) defines the genetic system and ensures system inheritance (Heng, 2009). Since the interaction of genes with the environment comprise the genetic system, and that most genes are neither independent information units nor common factors in disease, it is now easier to understand the importance of the stochastic genome alteration detected within various diseases. Stochastic genome alterations can no longer be considered insignificant noise as altered genomes yield altered networks (Heng, 2009; Heng et al., 2011a).

A key to appreciating the genome theory accepting the multiple level adaptive landscape model (Heng et al., 2011a,b, 2013a; Huang, 2013). In this model, pathway switching within a given cell represents microevolution, or small adaptation through local landscape change. In contrast, genome switching among cells often represents macroevolution or huge adaptation across the global landscape. Each genome-mediated global landscape can be achieved by large numbers of pathway-mediated local landscapes. Most of the current research on transcriptional re-programming in ER stress is likely focused on the local landscape level.

## STRESS-INDUCED GENOME DYNAMICS RESULT IN ADAPTATION AND DISEASE

Genome-level alterations are more effective at drastically changing the genetic system than gene mutation or epigenetic change, as supported by a recent study where karyotypic alterations were shown to influence gene expression profiles (Stevens et al., 2014). In addition, evidence in yeast studies strongly supports that aneuploidy directly affects gene expression, resulting in phenotypic variation (Pavelka et al., 2010). Genome-level alterations at the somatic cell level generate new systems by creating new frameworks, rather than new features defined by gene mutation/epigenetic regulation. Thus, genome alteration results in new genetic networks, suggesting that somatic cell genome evolutionary dynamics provide adaptive advantages for cells against stress. Further, genome diversity within normal, healthy tissues allows for complex organ function while providing the genome heterogeneity necessary to account for organ function-associated stress, such as liver-mediated blood detoxification. This realization is of high importance as genomic alterations were previously only viewed in a negative light.

Stochastic somatic genome dynamics can also result in disease onset and promotion. Higher NCCA frequencies have been linked with genome instability, disease conditions, and drug resistance (Heng et al., 2006, 2011a,b; Heng, 2007c, 2010; Ye et al., 2009). This realization provides explanation for the many common diseases that have not yet been linked with common biomarkers within the majority of cases. Focusing on genome alterations can unify the diverse factors that have been linked to individual genes by current molecular studies.

Therefore, adaptation requires “noise” elevation or an increase in heterogeneity. However, increased system dynamics can also potentially lead to disease onset. Now the question is, how does the bio-system solve this paradox of promoting system dynamics for short-term adaptation while avoiding the accumulation of alterations that could potentially harm the species?

This paradox was addressed by re-evaluation of the main function of sex. The century-old reasoning states that sexual reproduction functions to increase genetic variation. Under the new paradigm, sexual reproduction primarily acts to reduce genomic alterations despite its secondary function of mixing genes (Heng, 2007a; Wilkins and Holliday, 2009; Gorelick and Heng, 2011). Thus, sexual reproduction functions as a filter that effectively eliminates high-levels of stochastic genome alterations. This relationship between stochastic somatic genome dynamics and genome purification through sexual reproduction solves the conflict between short-term dynamics of adaptation (for somatic cell function) and long-term system persistence (to preserve the species). Stochastic somatic genome-level aberrations provide individuals with an evolutionary advantage against stress. However, somatic genomic aberrations could also lead to the onset and progression of common disease. In contrast to increasing evolutionary potential by stochastic somatic genomic aberrations, the constraint of germ line evolution through sexual reproduction preserves system integrity. Through this separation of germ line and somatic cell genomes, somatic genome alteration ensures short-term adaptation, while the filtering process of the germ line genome ensures long-term genome system identity (Horne et al., 2013a; Heng, 2014).

## CONCLUSION AND FUTURE PERSPECTIVE

By reviewing the importance of genome alteration in somatic evolution and its potential link with stress, we hope readers can grasp the rationale of studying the genome rather than pathways and understand the key relationship between stress, adaptation advantages, and the evolutionary trade-off. Even though refocusing on genome changes seems counterintuitive, as the resolution is lower at the genome-level than gene and pathway levels, it is the genome package that serves as the evolutionary selection unit in somatic cell evolution, especially in pathological conditions. As pointed out by Barbara McClintock, “in the future, attention undoubtedly will be centered on the genome…a highly sensitive organ of the cell that…senses unusual and unexpected events, and responds to them, often by restructuring the genome” (McClintock, 1984).

New strategies need to be developed to monitor system behavior under stress. More attention is needed to study the linkage of chromosomal instability (CIN) with various types of stress, as CIN serves as a general mechanism for cancer and potentially for other common diseases (Heng, 2010; Burrell et al., 2013; Heng et al., 2013a). In addition to our studies that link many stresses to CIN, ER stress has been directly linked to chromosome maintenance (Henry et al., 2010) and aneuploidy status (Sheltzer et al., 2012). Significantly, the linkage of stress-induced genome chaos (the survival strategy induced under high-levels of stress) and molecular pathway diversity have illustrated the ultimate importance of genome re-organization in cancer (Liu et al., 2014; Stevens et al., 2014).

Fortunately, the level of stochastic genomic change is the best measuring tool. Applying single cell-based approaches to measure the population profile is key (Heng et al., 2006; Abdallah et al., 2013). Although common practice may include disregarding these “noisy” data, heterogeneity in fact provides the complexity necessary for organismal survival and adaptation after stress, thus these data are of ultimate importance (Heng, 2014). Further studies are urgently needed to compare genome-level measurements with other known methods that focus on gene or pathway levels (Tang and Amon, 2013). Despite the complexity, measuring higher-level behaviors could be simpler than measuring lower-level diversity. For example, the family history of heart disease (higher-level of phenotype) has much more prediction power than comparing individual molecular markers (Heng, 2013).

A more systematical view is needed when dealing with stress and response. We need to monitor how the genome system changes during evolution, rather than only focus on specific pathways (using linear models) within limited time scales. By drastically simplifying the system to eliminate the heterogeneity, we might be able to identify an artificial linear relationship, but this often does not reflect clinical reality where multiple levels of heterogeneity rule. To change this situation, this discussed genome-mediated evolutionary concept must be incorporated into the field of stress research.

## AUTHOR CONTRIBUTIONS

Wrote the manuscript: Steven D. Horne and Henry H. Q. Heng. Participated in planning and editing: Saroj K. Chowdhury.

## Conflict of Interest Statement

The authors declare that the research was conducted in the absence of any commercial or financial relationships that could be construed as a potential conflict of interest. The Guest Associate EditorKezhong Zhang declares that, despite being affiliated to the same institution as authors Steven D. Horne and Henry H. Q. Heng, the review process was handled objectively and no conflict of interest exists.

## Abbreviations

CCAs: clonal chromosome aberrations
CIN: chromosomal instability
NCCAs: non-clonal chromosome aberrations
UPR: unfolded protein response
